# Dynamic coopetition game between private label and national brand under carbon trading policy

**DOI:** 10.1016/j.heliyon.2023.e14348

**Published:** 2023-03-08

**Authors:** Wenfang Yu, Guisheng Hou, Yu He, Baogui Xin

**Affiliations:** a College of Economics and Management, Shandong University of Science and Technology, Qingdao, 266590, China; b College of International Exchange, Shandong University of Science and Technology, Qingdao, 266590, China

**Keywords:** Dynamic coopetition game, Coopetition differential game, Private-label (PL) retailer, National-brand (NB) manufacturer, Carbon trading policy

## Abstract

The increasing environmental concerns have changed the coopetition behavior between private-label (PL) retailers and national-brand (NB) manufacturers, which needs firms to reconsider their competitive and cooperative strategies to cope with this change. To address this need, we incorporate carbon trading policy into dynamic coopetition game models between a PL retailer and an NB manufacturer. We examine their dynamic evolution trajectories of state variables, decision variables, and profit value functions by using numerical simulation. The sensitivity analysis shows that: (i) some key parameters affect the steady-state values of variables and profit value functions; (ii) the relative strengths of the NB manufacturer and the PL retailer affect decision variables and profit value functions. The results indicate that the carbon trading policy significantly affects dynamic coopetition between the PL retailer and the NB manufacturer.

## 1 Introduction

Store brands are defined as products that are developed, introduced into the market, stored, and promoted by retailers [1]. Because store brands carry a retailer's label name, they are now more popularly called private labels (PLs). PLs around the world show a positive market penetration. According to the PLMA's 2021 International Private Label Yearbook, PLs possess above 30% of the market in 16 European countries, nearly half of which hold market shares above 40%. In 2020, the growth rate of full-year sales for PLs exceeded national brands' full-year growth, which was 10.2%; in 2021, PL sales reached a new record of $199bn in all U.S. retail channels, according to the PLMA's 2022 Private Label Report. In China, the item number of PLs has increased substantially in the last three years, from China Private Label Development Research Report 2021. The considerable growth in PL sales has intensified competition between PL retailers and National Brand (NB) manufacturers, which poses severe challenges to NB manufacturers [2]. In general, most researchers and CEOs believe that PL retailers pose a substantial competitive threat to NB manufacturers, so much literature pays too much attention to their competitive relationship [3,4]. But in fact, PL retailers play a dual role as downstream partners of NB supply chains and brand competitors of NB manufacturers [5]. Thus, the dual role provides us with a meaningful perspective to study competitive and cooperative (coopetition) strategies between NB manufacturers and PL retailers.

Since the industrial revolution, the carbon dioxide and other greenhouse gases released into the atmosphere by human beings have increased year by year. The greenhouse effect has been enhanced, triggering a series of problems and attracting the attention of countries worldwide [6]. Governments and enterprises have proposed emission reduction targets and commitments to curb further global warming and the deterioration of environmental issues [[7], [8], [9]]. The carbon trading market is gradually taking shape. Carbon trading combines the scientific issue of climate change and the technical problem of reducing carbon emissions with the economic situation of sustainable development and solves the comprehensive scientific, technological, and economic integration problem by market mechanism [10,11]. Carbon emission reduction has become an essential element that cannot be ignored in the decision process of enterprises. However, little research focuses on carbon trading in the dynamic coopetition decision process, including the coopetition differential game [[12], [13], [14], [15], [16]]. This paper is intended to study the coopetition differential game between NB manufacturers and PL retailers under the carbon trading policy.

The possible novelties of this study are as follows.

(i) The proposed dynamic coopetition game enriches the existing competitive and cooperative theories. In this study, two mutually exclusive games, competitive and cooperative differential games, are merged into a unified differential game, a coopetition differential game.
(ii) A carbon trading policy is integrated into the coopetition differential game. The existing studies proved that a carbon trading policy can enrich competitive and cooperative differential game theories. However, how the carbon trading policy affects the coopetition differential game between NB manufacturers and PL retailers still needs to be studied.

The remainder of this paper is organized as follows. Section 2 briefly reviews the relevant literature. Section 3 defines the problem and proposes some assumptions. Section 4 builds a coopetition differential game model and presents its solutions. Section 5 implements some numerical simulations and sensitivity analysis. Section 6 provides conclusions and suggestions for future research.

## 2 Literature review

As an important domain for industrial practice, coopetition is the use of both cooperation and competition to enlarge a market and that produces win-win outcomes for both sides [[14], [15], [16], [17]]. Bengtsson & Kock [13] suggested that coopetition should be a paradoxical relationship between two or more players simultaneously involved in cooperative and competitive interactions. We structure our literature review from three relevant research streams, including (i) dynamic coopetition game model, (ii) competitive and cooperative games between PL retailers and NB manufacturers, and (iii) carbon trading in supply chains.

### 2.1 Dynamic coopetition game model

There is a lot of literature on dynamic coopetition game models, which can be mainly divided into two catalogs: coopetition differential games, other dynamic coopetition games including coopetition evolutionary games, and dynamic coopetition oligopoly games. The former includes many research results, such as a coopetition duopoly differential game licensing to a competitor [18], and a blockchain-based coopetition differential game for cloud computing [19]. The latter also includes many research results, such as a dynamic coopetition game between power supply enterprises [20], a dynamic coopetition game for cognitive radio spectrum sharing scheme [21], a dynamic Cournot game in a coopetition supply chain [22], a coopetitive duopoly game with implicit collusion [23], a dynamic coopetition game of renewable energy trade between China and the ‘Belt and Road’ countries , a dynamical coopetitive agreement of competitors within a specific eco-industrial park [24]. In this study, we will borrow some ideas from the literature mentioned above to build the following coopetition differential game model. The differential game not only can accurately describe the changing process of a system state, but also can effectively analyze the strategic decisions of the two players in continuous time. Therefore, this method is very consistent with our research content, and enhances the timeliness and accuracy of the dynamic coopetition game of both sides in this paper.

### 2.2 Competitive and cooperative games between PL and NB

PL retailers play a dual role as downstream partners of NB supply chains and competitors of NB manufacturers. PL and NB products are different due to different manufacturers. According to the quality of PL products, Kumar et al. [25] classified PLs into three types: Economy Private Label (EPL), Standard Private Label (SPL), and Premium Private Label (PPL). Compared with NB products, EPL, SPL, and PPL products are at low, medium, and high levels in terms of quality and price, respectively.

Throughout the existing literature on the games between PL retailers and NB manufacturers, studies on EPL and PPL mainly focus on price games [2]. Chan Choi [26] developed a price-game model between an NB manufacturer and a PL retailer and pointed out that establishing brand premium should be the first protection measure for the NB manufacturer rather than drastically reducing wholesale prices. Huang & Feng [27] studied a price game between an NB manufacturer and a PL retailer with different money-back guarantee policies. Karray & Martín-Herrán [28] described competition between NBs and PLs as a consumer-utility-function game model considering diverse decision timing choices of pricing and advertising. They drew the following conclusions: the NB manufacturers sustain losses because of the introduction of PLs under unchanged decision timing, but the NB manufacturers can benefit from or prevent losses by changing their decision timing. There is a lot of literature from different perspectives on multilateral contracting [29], market expansion and bargaining power [30], price transmission [31], market power and pricing actions [32] to discuss price competition between NB manufacturers and PL retailers.

Another vital game perspective is quality. Chakraborty et al. [33] examined the quality competition between a PL retailer and an NB manufacturer. They pointed out that the quality level of PL products is higher than that of NB products if there is no cost variance. Still, the retail price of PL products is lower than that of NB products, and the quality does not affect the retail price. Zhu et al. [34] studied effect of quality differences between NB and PL products on the equilibrium results and the NB manufacturer's profits. They concluded that an enormous quality difference enhances the economic benefit of the NB manufacturer. Many scholars explicated quality competition between NB manufacturers and PL retailers from different directions, such as quality orientation under sourcing structures [35], NB quality's positive effect on NB manufacturers' sourcing [36], channel strategy for lower-quality PL products [37], PL product with sufficiently low quality under symmetric spillover rate [38], optimal quality position of PL products [39].

The above literature focuses on price and quality game of differentiated products between NB manufacturers and PL retailers, while output game is rarely involved. In the real-world market, many powerful PL retailers, such as Metro and Wal-Mart, produce PL products with no difference compared with NB products in terms of quality and price. Given market share, the encroachment of undifferentiated PL products will dilute the original market share of NB products. In addition, there are many uncertainties in the process of PL products' entry into the NB market. NB manufacturers and PL retailers must adjust their output according to market changes to avoid substantial business losses. Therefore, in the case of no difference in products, the output game between NB manufacturers and PL retailers is of great practical significance. This is also the starting point of this study.

### 2.3 Carbon trading in supply chains

To effectively control the greenhouse effect, carbon abatement has become a consensus of the world. Since the Kyoto Protocol came into effect in 2005, the global carbon trading market has exploded, and carbon trading has attracted significant concerns from researchers. Shen et al. [40] reviewed the carbon trading literature and divided the research field into three phases: design of carbon trading schemes (1998–2006), carbon price-related issues (2007–2011), and interaction between carbon trading schemes and the economy (2012-as yet).

As countries around the world set emission reduction targets, carbon trading has become an important issue that must be considered for social and economic development. The effect of carbon trading on supply chain decisions is drawing more and more attention, and current research mainly focuses on three aspects.

The first is the carbon trading policy's effect on manufacturers' competition. For example, Huang et al. [41] built a duopoly model comprising two competing manufacturers to discuss the impact of carbon trading on manufacturer transformation. Meng et al. [42] conducted a remanufacturing competition system consisting of an original equipment manufacturer and an independent remanufacturer. Wang et al. [43] investigated the impact of carbon trading policy and consumers' low-carbon preferences on the manufacturers' product lines. Xia et al. [10] and Liao et al. [44] discussed the effect of carbon trading on remanufacturers.

The second is the carbon trading policy's effect on supply chains. Li et al. [45] incorporated the carbon trading mechanism into cooperative decisions of competitive supply chains through the Stackelberg game. Wang et al. [46] considered different carbon trading mechanisms in a global supply chain for production planning with transshipment with a mixed integer programming model. Cheng et al. [47] studied carbon cap-and-trade in a closed-loop supply chain through a non-cooperative game theory model. They proved that carbon emission limitation might incentivize manufacturers to increase investment in green technology. Yang et al. [48] conducted game theoretic models to examine the different behavior impacts when players do not comply with the carbon cap-and-trade scheme. Some scholars combined carbon trading policy with other factors to explore joint influences on supply chains, for instance, carbon cap-and-trade regulation and socially responsible behaviors [49], carbon trading mechanism and sustainable development [50], carbon trade and trade credit [51], social welfare and cap-and-trade regulation [52].

The third is the carbon trading policy's effect on competition among supply chain members. Xue et al. [52] established and solved game models between a manufacturer and a retailer and investigated the retailer's incentive strategies to increase the manufacturer's motivation for carbon emission reduction. Yang & Gao [53] took into account carbon trading policy and consumers' dual preferences simultaneously to discuss the equilibrium decision-making problem in a two-level fresh agricultural products supply chain. Wang et al. [54] incorporated cap-and-trade regulations and consumers' low-carbon preferences into differential game models to examine the emission reduction decisions of a manufacturer and a supplier. Zhang et al. [55] considered a cap-and-trade mechanism and trade credits in decentralized and centralized models. Sun & Yang [56] considered consumers' environmental awareness and developed Cournot and collusion competitive models to explore optimal carbon emission reduction decisions for two competitive manufacturers.

Combining the existing literature from three aspects, we find that carbon trading policy is more straightforwardly associated with manufacturers, especially remanufacturers because environmental requirements are challenging. The competitive ability of supply chains is increasingly influenced by carbon trading policy, including closed-loop and global supply chains. Carbon trading policy also affects competition among supply chain members, such as manufacturers, retailers, and suppliers. In China, Beijing, Shanghai, and Shenzhen have included retail enterprises in their local carbon trading market. In October 2022, the powerful retailer SKP announced that it had achieved full carbon neutrality in Beijing SKP, Xi'an SKP, and SKP-S (Beijing) by purchasing carbon allowances and CCER. Researchers combine carbon trading policy with many effect factors, such as carbon cap-and-trade regulation, socially responsible behaviors, sustainable development, social welfare, consumers' preferences, *etc*. However, there is little literature on dynamic coopetition between PL retailers and NB manufacturers under carbon trading policy.

With the proposal of carbon peaking and carbon neutrality goals, carbon emission reduction is still a vital link that must be addressed for the production process. Under the carbon emission reduction policy, NB manufacturers and PL retailers need to play the output game with each other and face the urgent dilemma between their outputs and carbon emission reductions. Thus, this study will establish a dynamic coopetition game model between a PL retailer and an NB manufacturer under a carbon trading policy to optimize both sides' outputs and carbon emission reductions.

## 3 Dynamic coopetition game model

To further clarify the coopetition game, let's analyze the following real-world example of Roku TV brand competition and cooperation between TCL Company and Walmart Company. Roku TV is a home appliance with two primary brands: TCL and Onn. The TCL is a national brand of Roku TV, but Onn is a private brand of Roku TV, Walmart's leading tech brand. The TCL Company is a professional manufacturer of Roku TVs. Walmart is an experienced retailer with two identities: the retailer of Roku TV of TCL and the manufacturer and retailer of Roku TV of Onn.

Regarding Roku TV of TCL, there is a supply and marketing relationship between TCL and Walmart, which is mainly cooperative. When Walmart produces and sells its Roku TV of Onn, Walmart also needs carbon emission rights. On the one hand, Onn and TCL products will compete in satisfying customer demands; On the other hand, Walmart and TCL will have purchase competition in the carbon emission market. Thus, we will design a coopetition game as follows.

### 3.1 Conceptual framework

To facilitate subsequent expression, the superscript ‘N’ indicates the dynamic coopetition game between a PL retailer and an NB manufacturer under carbon trading policy. Under the carbon trading policy, enterprises calculate their carbon emission rights that can be traded or need to be purchased according to their initial carbon emission quotas, carbon emissions, and emission reductions. For enterprises with surplus carbon emission rights, carbon trading volume will be included in their profits. Conversely, for enterprises with a shortage of carbon emission rights, the carbon trading volume will be deducted as a cost from their profits. Therefore, enterprises are motivated to implement carbon emission reduction behaviors under the carbon trading policy. However, carbon emission is often proportional to production in the production process, and carbon emission reduction behavior will incur corresponding costs. It is an urgent dilemma for the PL retailer and the NB manufacturer to balance their production and carbon emission reduction.

**Fig. 1:**
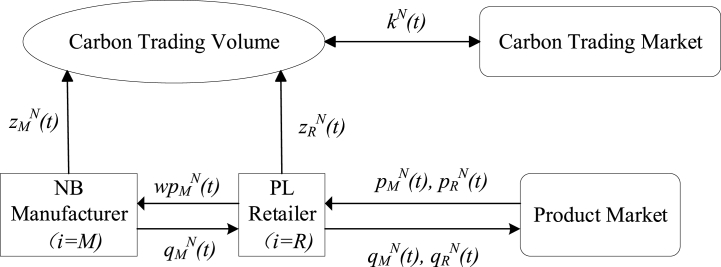
Conceptual framework of the dynamic coopetition game.

This paper studies a two-echelon supply chain consisting of an NB manufacturer (i=M) and a PL retailer (i=R), as shown in Fig. 1. On the one hand, the NB manufacturer wholesales NB products whose output is ${q_{M}}^{N}\left(t\right)$, to the PL retailer at a part price $w{p_{M}}^{N}\left(t\right)$ （0<w<1）of the retail price ${p_{M}}^{N}\left(t\right)$, and then the PL retailer sells the NB products to consumers at the retail price ${p_{M}}^{N}\left(t\right)$. The NB manufacturer and the PL retailer are cooperative on this point. On the other hand, meanwhile, the PL retailer introduces PL products with the same quality as NB products into the market, whose output is ${q_{R}}^{N}\left(t\right)$, and sells them to consumers at retail price ${p_{R}}^{N}\left(t\right)$. Two homogeneous products compete for market share, and different market shares naturally affect their outputs. The NB manufacturer and the PL retailer constitute a competitive relationship on this point. In addition, the NB manufacturer and the PL retailer need to decide their carbon emission reductions ${z_{i}}^{N}\left(t\right)$ in the production process and trade carbon emission rights at a price $k^{N}\left(t\right)$ in the carbon trading market, according to their individual needs. Based on the above, this study analyzes the output game between the NB manufacturer and the PL retailer under the carbon trading policy, and the dynamic decision optimization of outputs and emission reductions.

### 3.2 Assumption

The dynamic coopetition game model between the PL retailer and the NB manufacturer under the carbon trading policy is assumed as follows.

(i) Similar to Xin & Sun [9] and Wu [18], the carbon trading price $k^{N}\left(t\right)$ keeps adjusting with time t, and is influenced by the government guiding price $\bar{k}$, so the dynamic evolution equation of $k^{N}\left(t\right)$ follows a first-order adjustment process as follows:

(1) $${\dot{k}}^{N}\left(t\right)=\alpha_{k}\left(\bar{k}-k^{N}\left(t\right)\right),k\left(0\right)=k_{0}$$

where, $\alpha_{k}>0$ represents the adjustment coefficient of the carbon trading price, and $k_{0}>0$ represents the initial carbon trading price.

(ii) Similar to Xin & Sun [9], the expression of final carbon trading volumes of the NB manufacturer and the PL retailer is ${A_{i}}^{N}\left(t\right)=B_{i}-\beta_{i}{q_{i}}^{N}\left(t\right)-{z_{i}}^{N}\left(t\right)$, where, $B_{i}>0$ represents the initial carbon emission right quota of enterprise i, $\beta_{i}\geq 0$ represents the carbon emission generated by the unit output of enterprise i, ${q_{i}}^{N}\left(t\right)$ represents the output of enterprise i, ${z_{i}}^{N}\left(t\right)$ represents the carbon emission reduction of enterprise i.
(iii) Following Corbett & Karmarkar [57], since NB and PL products are homogeneous, their retail prices ${p_{i}}^{N}\left(t\right)$ are respectively set as ${p_{i}}^{N}\left(t\right)=h_{i}-\sum {q_{i}}^{N}\left(t\right)$, where $h_{i}$ indicates the price ceiling of the product i.
(iv) Following Zu et al. [58], the NB manufacturer sells its product to the PL retailer at wholesale price $w{p_{M}}^{N}\left(t\right)$, where 0<w<1 represents the rate of return per unit product of the NB manufacturer.
(v) Similar to Xia et al. [59], Sun & Yang [56], and Wang et al. [54], the cost of enterprise's emission reduction is assumed to be a quadratic function ${C_{i}}^{N}\left(t\right)=\frac{1}{2}\mu_{i}{\left({z_{i}}^{N}\left(t\right)\right)}^{2}$, where $\mu_{i}>0$ represents emission reduction cost coefficient of enterprise i, which reflects the enterprise's emission reduction efficiency. The smaller the $\mu_{i}$, the higher the emission reduction efficiency.

### 3.3 Model

Based on the conceptual model description and assumptions in Section 3, the profit functions of the NB manufacturer and the PL retailer are expressed as follows.

(2) $$\pi_{M}^{N}\left(t\right)=wp_{M}^{N}\left(t\right)q_{M}^{N}\left(t\right)-C_{M}\left(t\right)+k^{N}\left(t\right)A_{M}\left(t\right)$$

(3) $$\pi_{R}^{N}\left(t\right)=\left(1-w\right)p_{M}^{N}\left(t\right)q_{M}^{N}\left(t\right)+p_{R}^{N}\left(t\right)q_{R}^{N}\left(t\right)-C_{R}\left(t\right)+k^{N}\left(t\right)A_{R}\left(t\right)$$

Similar to Xin & Sun [9] and Zu et al. [58], the following optimization problems of the NB manufacturer and the PL retailer can be described according to Eq. (1)∼(3).

(4) $$\begin{array}{l} \max_{q_{M}^{N}\left(t\right),z_{M}^{N}\left(t\right)}J_{M}^{N}\left(t\right)={}_{\int }^{\infty }0\;e^{-rt}\pi_{M}^{N}\left(t\right)dt\text{,}\\ s.t.\;\begin{array}{cc} {\dot{k}}^{N}\left(t\right)=\alpha_{k}\left(\bar{k}-k^{N}\left(t\right)\right), & k\left(0\right)=k_{0} \end{array}\text{,} \end{array}$$

(5) $$\begin{array}{l} \max_{q_{R}^{N}\left(t\right),z_{R}^{N}\left(t\right)}J_{R}^{N}\left(t\right)={}_{\int }^{\infty }0\;e^{-rt}\pi_{R}^{N}\left(t\right)dt\text{,}\\ s.t.\;\begin{array}{cc} {\dot{k}}^{N}\left(t\right)=\alpha_{k}\left(\bar{k}-k^{N}\left(t\right)\right), & k\left(0\right)=k_{0} \end{array}\text{,} \end{array}$$

where $J_{M}^{N}\left(t\right)$ and $J_{R}^{N}\left(t\right)$ represent the net discounted value functions of the NB manufacturer and the PL retailer, respectively. r represents the discount rate.

## 4 Optimal solution

### 4.1 Feedback solution

In this section, we will search for the optimal feedback solutions [9,60] for the above models. Without confusion, the time variable t will be omitted later in this paper.

First, the Hamilton functions of Eq. (4)∼(5) are respectively expressed as follows.

(6) $$rV_{M}^{N}=\max_{q_{M}^{N},z_{M}^{N}}\left\{\pi_{M}^{N}+{\dot{k}}^{N}V_{Mk}^{N}\right\}\text{,}$$

(7) $$rV_{R}^{N}=\max_{q_{R}^{N},z_{R}^{N}}\left\{\pi_{R}^{N}+{\dot{k}}^{N}V_{Rk}^{N}\right\}\text{,}$$

where $V_{M}^{N}=V_{M}^{N}\left(k^{N}\right)$ and $V_{R}^{N}=V_{R}^{N}\left(k^{N}\right)$ indicate profit value functions of the NB manufacturer and the PL retailer, respectively. $V_{Mk}^{N}=\frac{\partial V_{M}^{N}}{\partial k^{N}}$ and $V_{Rk}^{N}=\frac{\partial V_{R}^{N}}{\partial k^{N}}$ represent the partial derivatives of profit value functions of the NB manufacturer and the PL retailer with respect to state variable $k^{N}$, respectively.

Solving the first order partial derivatives with respect to carbon emission reductions and outputs of the NB manufacturer and the PL retailer in Eq. (6)∼(7), we can obtain

(8) $$\left\{ \begin{array}{c} k^{N}-\mu_{M}z_{M}^{N}=0\text{,}\\ \left(h_{M}-2q_{M}^{N}-q_{R}^{N}\right)w-k^{N}\beta_{M}=0\text{,}\\ k^{N}-\mu_{R}z_{R}^{N}=0\text{,}\\ h_{R}-2q_{R}^{N}-\left(2-w\right)q_{M}^{N}-k^{N}\beta_{R}=0\text{.} \end{array}\right.$$

Solving Eq. (8), we can get the optimal feedback solutions of carbon emission reductions and outputs of the NB manufacturer and the PL retailer as follows.

(9) $$z_{M}^{N*}=\frac{k^{N}}{\mu_{M}}$$

(10) $$q_{M}^{N*}=\frac{w\left(2h_{M}-h_{R}\right)+k^{N}\left(w\beta_{R}-2\beta_{M}\right)}{w\left(2+w\right)}\text{,}$$

(11) $$z_{R}^{N*}=\frac{k^{N}}{\mu_{R}}\text{,}$$

(12) $$q_{R}^{N*}=\frac{2h_{R}w-h_{M}w\left(2-w\right)+k^{N}\left(2\beta_{M}-w\left(\beta_{M}+2\beta_{R}\right)\right)}{w\left(2+w\right)}$$

Proposition 1 Under the carbon trading policy, the evolution track of the optimal carbon trading price over time is the following Eq. (13).

(13) $${k^{N}}^{\infty }=\bar{k}+C_{1}e^{-\alpha_{k}t}$$

*where* $C_{1}=k_{0}-\bar{k}$.

Proposition 2 *Under the carbon trading policy*, *the evolution tracks of the optimal carbon emission reductions and outputs of the NB manufacturer and the PL retailer over time are the following* Eq. (14)*∼*(17).

(14) $$z_{M}^{N\infty }=\frac{{k^{N}}^{\infty }}{\mu_{M}}$$

(15) $$q_{M}^{N\infty }=\frac{w\left(2h_{M}-h_{R}\right)+{k^{N}}^{\infty }\left(w\beta_{R}-2\beta_{M}\right)}{w\left(2+w\right)}$$

(16) $$z_{R}^{N\infty }=\frac{{k^{N}}^{\infty }}{\mu_{R}}$$

(17) $$q_{R}^{N\infty }=\frac{2h_{R}w-h_{M}w\left(2-w\right)+{k^{N}}^{\infty }\left(2\beta_{M}-w\left(\beta_{M}+2\beta_{R}\right)\right)}{w\left(2+w\right)}$$

Proposition 3 *Under the carbon trading policy*, *the evolution tracks of the optimal profit value functions of the NB manufacturer and the PL retailer over time are as follows*.

(18) $$V_{M}^{N\infty }=m_{0}^{N}+m_{1}^{N}{k^{N}}^{\infty }+m_{2}^{N}{\left({k^{N}}^{\infty }\right)}^{2}$$

(19) $$V_{R}^{N\infty }=n_{0}^{N}+n_{1}^{N}{k^{N}}^{\infty }+n_{2}^{N}{\left({k^{N}}^{\infty }\right)}^{2}$$

*where*

$$\left\{ \begin{array}{c} m_{0}^{N}=\frac{{\left(h_{R}-2h_{M}\right)}^{2}w+\alpha_{k}\bar{k}m_{1}^{N}{\left(2+w\right)}^{2}}{r{\left(2+w\right)}^{2}}\text{,}\\ m_{1}^{N}=\frac{B_{M}{\left(2+w\right)}^{2}-\alpha_{k}\left(m_{1}^{N}-2\bar{k}m_{2}^{N}\right){\left(2+w\right)}^{2}+4\beta_{M}\left(h_{R}-2h_{M}\right)+2w\beta_{R}\left(2h_{M}-h_{R}\right)}{r{\left(2+w\right)}^{2}}\text{,}\\ m_{2}^{N}=\frac{w{\left(2+w\right)}^{2}-2\left(2\alpha_{k}wm_{2}^{N}{\left(2+w\right)}^{2}-{\left(-2\beta_{M}+w\beta_{R}\right)}^{2}\right)\mu_{M}}{2rw{\left(2+w\right)}^{2}\mu_{M}}\text{,}\\ n_{0}^{N}=\frac{{h_{R}}^{2}\left(3+w\right)+h_{M}h_{R}\left(w+w^{2}-6\right)-{h_{M}}^{2}\left(w\left(2+w\right)-4\right)+\alpha_{k}\bar{k}n_{1}^{N}{\left(2+w\right)}^{2}}{r{\left(2+w\right)}^{2}}\text{,}\\ n_{1}^{N}=\frac{4\left(h_{R}-h_{M}\right)\beta_{M}+w\left({\left(2+w\right)}^{2}\left(B_{R}-\alpha_{k}n_{1}^{N}+2\alpha_{k}\bar{k}n_{2}^{N}\right)+2h_{M}\beta_{M}-\beta_{R}\left(3+w\right)\left(2h_{R}-h_{M}\left(2-w\right)\right)\right)}{rw{\left(2+w\right)}^{2}}\text{,}\\ n_{2}^{N}=\frac{w{\left(2+w\right)}^{2}+2\mu_{R}\left(w{\beta_{M}}^{2}-2\alpha_{k}wn_{2}^{N}{\left(2+w\right)}^{2}-4\beta_{M}\beta_{R}+w\left(3+w\right){\beta_{R}}^{2}\right)}{2rw{\left(2+w\right)}^{2}\mu_{R}}\text{.} \end{array}\right.$$

Proof Solving Eq. (1), the evolution track of the optimal carbon trading price over time can be obtained, as shown in Proposition 1. Substituting Eq. (13) into Eq. (9)∼(12), the evolution tracks of the optimal carbon emission reductions and outputs of the NB manufacturer and the PL retailer over time are obtained, as shown in Proposition 2.

*Substituting* Eq. (9)∼(12) *into* Eq. (6)*∼*(7), *we can obtain*

(20) $$rV_{M}^{N}=\max_{q_{M}^{N},z_{M}^{N}}\left\{\begin{array}{l} \frac{\left(w\left(2h_{M}-h_{R}\right)+k^{N}\left(w\beta_{R}-2\beta_{M}\right)\right)\left(2h_{M}-h_{R}+k^{N}\left(\beta_{M}+\beta_{R}\right)\right)}{{\left(2+w\right)}^{2}}-\frac{{\left(k^{N}\right)}^{2}}{2\mu_{M}}\\ +k^{N}\left(B_{M}-\frac{\beta_{M}\left(w\left(2h_{M}-h_{R}\right)+k^{N}\left(w\beta_{R}-2\beta_{M}\right)\right)}{2w+w^{2}}+\frac{k^{N}}{\mu_{M}}\right)+\alpha_{k}\left(\bar{k}-k^{N}\right)V_{Mk}^{N} \end{array}\right\}\text{,}$$

(21) $$rV_{R}^{N}=\max_{q_{R}^{N},z_{R}^{N}}\left\{\begin{array}{l} \frac{\left(1-w\right)\left(w\left(2h_{M}-h_{R}\right)+k^{N}\left(w\beta_{R}-2\beta_{M}\right)\right)\left(2h_{M}-h_{R}+k^{N}\left(\beta_{M}+\beta_{R}\right)\right)}{w{\left(2+w\right)}^{2}}-\frac{{\left(k^{N}\right)}^{2}}{2\mu_{R}}\\ +\frac{\left(h_{R}\left(1+w\right)-h_{M}w+k^{N}\left(\beta_{M}+\beta_{R}\right)\right)\left(2k^{N}\beta_{M}+w\left(2h_{R}-h_{M}\left(2-w\right)-k^{N}\left(\beta_{M}+2\beta_{R}\right)\right)\right)}{w{\left(2+w\right)}^{2}}\\ +k^{N}\left(B_{R}-\frac{\beta_{R}\left(2k^{N}\beta_{M}+w\left(2h_{R}-h_{M}\left(2-w\right)-k^{N}\left(\beta_{M}+2\beta_{R}\right)\right)\right)}{w\left(2+w\right)}+\frac{k^{N}}{\mu_{R}}\right)+\alpha_{k}\left(\bar{k}-k^{N}\right)V_{Rk}^{N} \end{array}\right\}$$

*According to* Eq. (20)∼(21), *we assume that the profit value functions of the NB manufacturer and the PL retailer are quadratic functions of carbon trading price* $k^{N}$, *as shown in* Eq. (18)∼(19), *respectively*. *Then by solving the first-order partial derivatives with respect to* $k^{N}$ *in* Eq. (18)∼(), *we can get the following* Eq. (22)*∼*(23).

(22) $$V_{Mk}^{N}=m_{1}^{N}+2m_{2}^{N}k^{N}$$

(23) $$V_{Rk}^{N}=n_{1}^{N}+2n_{2}^{N}k^{N}$$

*Substituting* Eq. (9)∼(12) *and* (18) *into* Eq. (20), *and let the left and right sides of the formula be equal*, *we can obtain the expressions of* $m_{0}^{N}$, $m_{1}^{N}$ *and* $m_{2}^{N}$. *Similarly*, *substituting* Eq. (9)∼(12) *and* (19) *into* Eq. (21), *and let the left and right sides of the formula be equal*, *we can obtain the expressions of* $n_{0}^{N}$, $n_{1}^{N}$ *and* $n_{2}^{N}$. Proposition 3 *is proven*.

### 4.2 Numerical simulation

To present Proposition 1, Proposition 3 more intuitively by numerical simulation using Matlab software, we initialize the parameters involved in the models as follows. $h_{M}=7$, $h_{R}=6$, $\alpha_{k}=0.2$, $\bar{k}=4$, $B_{M}=10$, $B_{R}=8$, $\beta_{M}=0.5$, $\beta_{R}=0.5$, $\mu_{M}=0.9$, $\mu_{R}=1.2$, w=0.5, r=0.01, $k_{0}=1.5$.

Substituting the above parameter settings into Proposition 1, Proposition 3, we can obtain the evolution equations over time of variables and functions only considering the carbon trading policy.

$${k^{N}}^{\infty }\left(t\right)=4-2.5\times e^{-0.2t},z_{M}^{N\infty }\left(t\right)=4.44-2.78\times e^{-0.2t},q_{M}^{N\infty }\left(t\right)=0.4+1.5\times e^{-0.2t},z_{R}^{N\infty }\left(t\right)=3.33-2.08\times e^{-0.2t},q_{R}^{N\infty }\left(t\right)=1.2-0.5\times e^{-0.2t},V_{M}^{N\infty }\left(t\right)=4896.89+11.21\times e^{-0.4t}-169.1\times e^{-0.2t}\text{,}$$

$$V_{R}^{N\infty }\left(t\right)=4122.67+4.22\times e^{-0.4t}-122.06\times e^{-0.2t}\text{.}$$

**Fig. 2:**
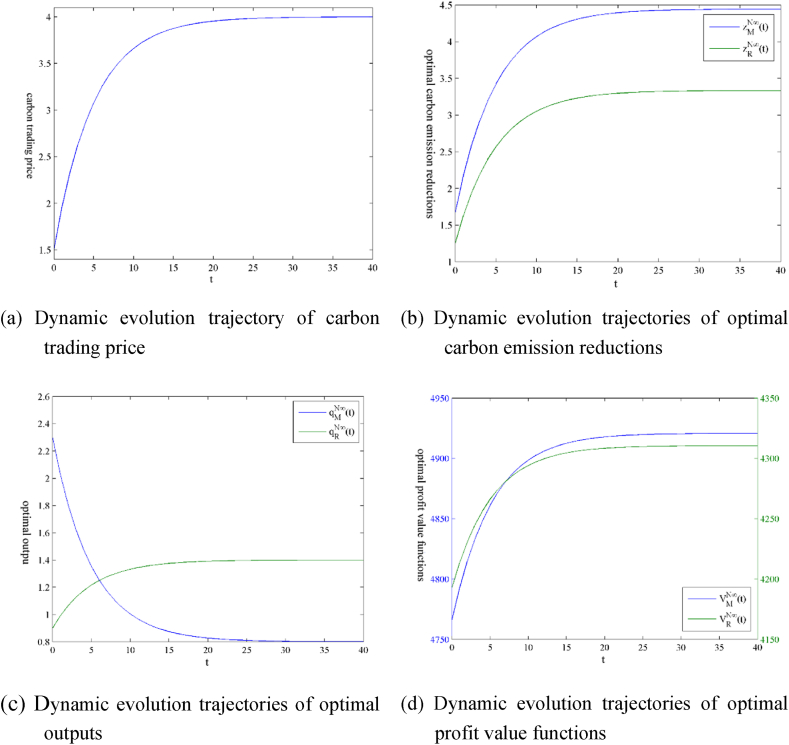
Dynamic evolution trajectories of variables and profit value functions under the scenario ‘N’.

Based on the above numerical expressions, Fig. 2 shows the dynamic evolution trajectories of state variables, decision variables, and profit value functions of the NB manufacturer and the PL retailer. Fig. 2(a) shows that the carbon trading price presents an uptrend first and then a steady state as time passes. Fig. 2(b) shows that the optimal carbon emission reductions of the NB manufacturer and the PL retailer are on rise first and then steady state, and the final carbon reduction amount of the NB manufacturer is more significant than that of the PL retailer. Fig. 2(c) shows that the optimal output of the NB manufacturer goes downward first and then presents a steady state over time, while the evolution trend of the optimal output of the PL retailer is the opposite, first in an uptrend and then a steady state. The NB manufacturer's output is greater than that of the PL retailer in the first six periods. However, the PL retailer's output exceeds that of the NB manufacturer after the 7^th^ period. Fig. 2(d) shows that, as time goes on, the optimal profit value functions of both the NB manufacturer and the PL retailer first increase and then stay in a steady state, and the final profit of the NB manufacturer exceeds that of the PL retailer.

The following sensitivity analysis will study the influence of each parameter on variables and profit value functions and explore the effects of relative strength statuses of the NB manufacturer and the PL retailer on the decision variables and profit value functions.

## 5 Sensitivity analysis

This section examines the following two aspects: (i) one is involved in the effects of some critical parameters on the steady-state values of variables and profit value functions in the models; (ii) the other is related to the effects of the relative strengths of the NB manufacturer and the PL retailer on decision variables and profit value functions.

### 5.1 Parameter sensitivity

**Fig. 3:**
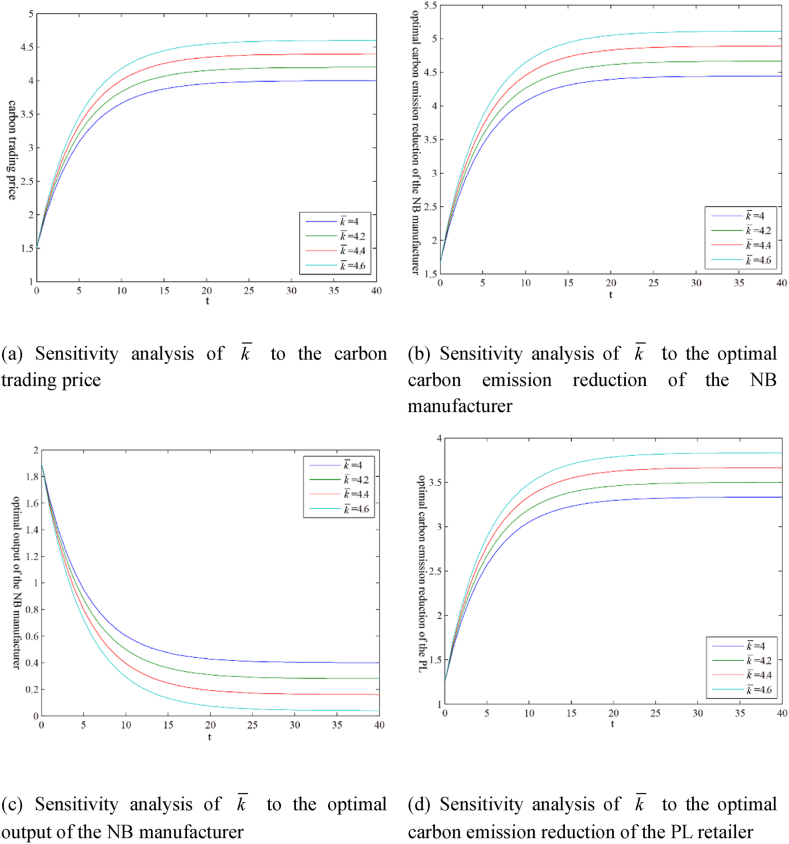

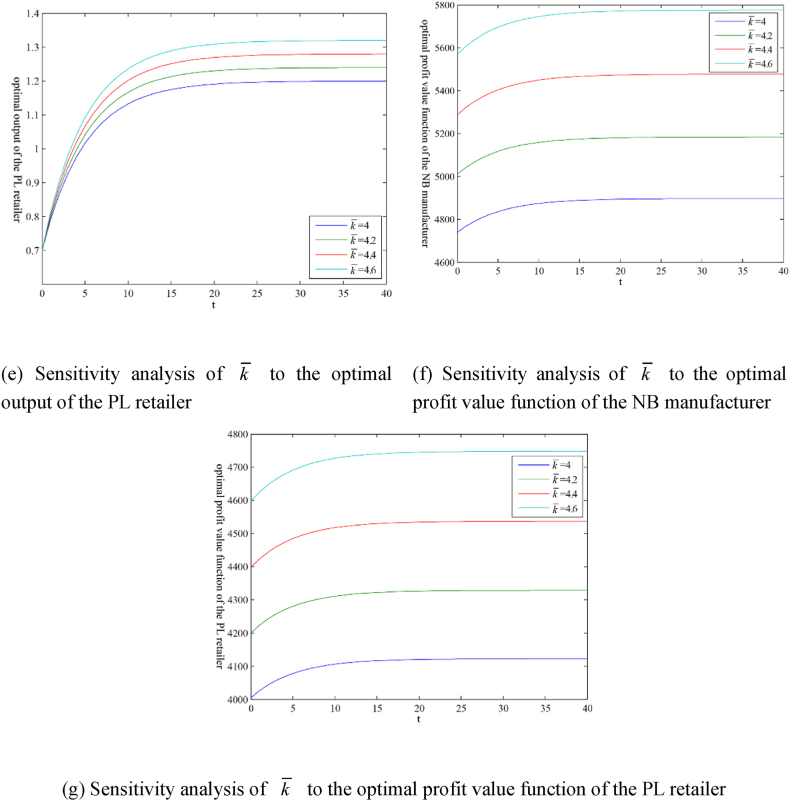
Sensitivity analysis of parameter $\bar{k}$ under the scenario ‘N’.

The models involve many parameters. Due to space limitations, this section only presents the guiding price $\bar{k}$ of carbon trading and the initial carbon trading price $k_{0}$ as numerical examples in the form of figures, as shown in Fig. 3(ãg) and 4 (ãg), respectively. Table 1 shows the sensitivity analysis results of other parameters. The superscript ‘ss’ indicates the steady-state value, that is, the value after variable convergence.

**Table 1:** Parameter sensitivity analysis under the scenario ‘N’.

	$k^{N,ss}$	$z_{M}^{N,ss}$	$q_{M}^{N,ss}$	$z_{R}^{N,ss}$	$q_{R}^{N,ss}$	$V_{M}^{N,ss}$	$V_{R}^{N,ss}$
$h_{M}$	/	/	+	/	–	+	*E*-L+
$h_{R}$	/	/	–	/	+	–	E− L+
$\bar{k}$	+	+	–	+	+	+	+
$B_{M}$	/	/	/	/	/	+	/
$B_{R}$	/	/	/	/	/	/	+
$\beta_{M}$	/	/	–	/	+	–	–
$\beta_{R}$	/	/	+	/	–	+	E− L+
$\mu_{M}$	/	–	/	/	/	–	/
$\mu_{R}$	/	/	/	–	/	/	–
w	/	/	+	/	–	+	E + L-
$k_{0}$	/	/	/	/	/	/	/

Note: ‘+’ indicates positive effect; ‘-’ indicates negative effect; ‘/’ indicates no effect; ‘E’ indicates earlier; ‘L’ indicates later.

Some results can be extracted from Table 1 as follows.

(i) For the carbon trading market, the only factor affecting the steady-state value $k^{N,ss}$ of the carbon trading price is the carbon trading guiding price $\bar{k}$.
(ii) For the NB manufacturer and the PL retailer, the factors that affect the steady-state value $z_{i}^{N,ss}$ of their carbon emission reductions include their emission reduction efficiencies $\mu_{i}$ and the carbon trading guiding price $\bar{k}$.
(iii) For the NB manufacturer, the factors that affect the output steady-state value $q_{M}^{N,ss}$ include the highest retail price $h_{i}$, the carbon trading guiding price $\bar{k}$, the carbon emission amount generated by unit output $\beta_{i}$, and the rate of return per unit NB product w. The factors that affect the steady-state value of the profit value function $V_{M}^{N,ss}$ include the highest retail price $h_{i}$, the carbon trading guiding price $\bar{k}$, the initial carbon emission right quota $B_{M}$, the carbon emission amount generated by unit output $\beta_{i}$, the emission reduction efficiency $\mu_{M}$ , and the rate of return per unit NB product w.
(iv) For the PL retailer, the factors affecting output steady-state value $q_{R}^{N,ss}$ are the same as for the NB manufacturer, but the effects of these factors on both are the opposite. The factors that affect the steady-state value of the profit value function $V_{R}^{N,ss}$ include the highest retail price $h_{i}$, the carbon trading guiding price $\bar{k}$, the initial carbon emission right quota $B_{R}$, the carbon emission amount generated by unit output $\beta_{i}$, the emission reduction efficiency $\mu_{R}$ , and the rate of return per unit NB product w.
(v) The highest retail price $h_{i}$, the carbon emission amount generated by unit PL product $\beta_{R}$ , and the rate of return per unit NB product w have effects with the tendency of ‘*E*-L+’ or ‘E + L-’ on the profit value function of the PL retailer. The main reason may be that these factors affect the market shares of PL and NB products.

**Fig. 4:**
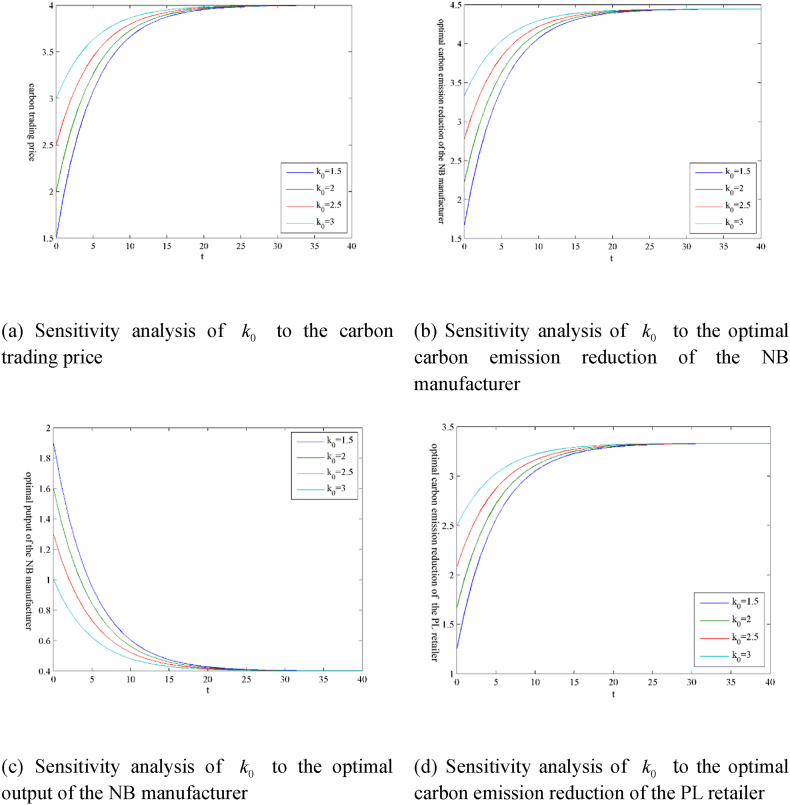

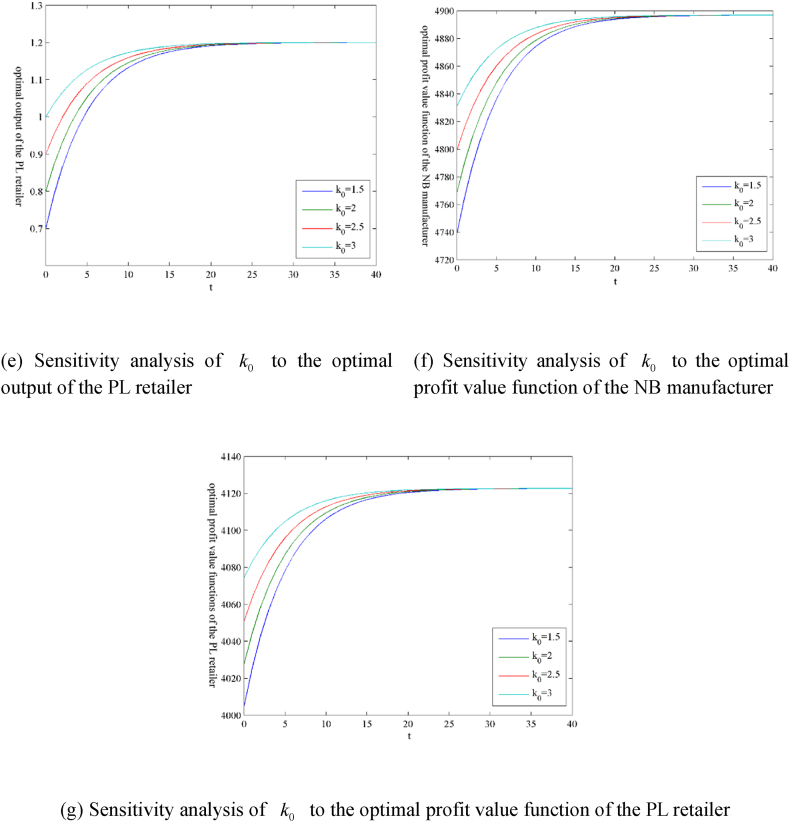
Sensitivity analysis of parameter $k_{0}$ under the scenario ‘N’.

In addition, the initial carbon trading price $k_{0}$ does not affect the steady-state values of the variables and the profit value functions. Still, it affects the convergence rates of the variables and the profit value functions, as shown in Fig. 4.

### 5.2 Sensitivity of the relative strength

Following the initial parameter settings in Section 3, we assume that the size relationship between the highest retail prices is $h_{M}\left(=7\right)>h_{R}\left(=6\right)$. It represents that consumers are willing to pay higher price for NB products than for PL products. That is to say, relative to the PL retailer, the NB manufacturer holds a higher status in the market. Fig. 2 shows the dynamic evolution results of related variables and profit value functions. In this section, we explore effects of the relative strengths of both the NB manufacturer and the PL retailer on the optimal decision variables and profit value functions by adjusting the size relationships of product maximum retail prices between the NB manufacturer and the PL retailer. If other parameters remain unchanged, it is assumed that $h_{M}\left(=7\right)=h_{R}\left(=7\right)$ represents that the NB manufacturer and the PL retailer are in the same status, and $h_{M}\left(=6\right)<h_{R}\left(=7\right)$ represents that the PL retailer holds a higher status than the NB manufacturer.

**Fig. 5:**
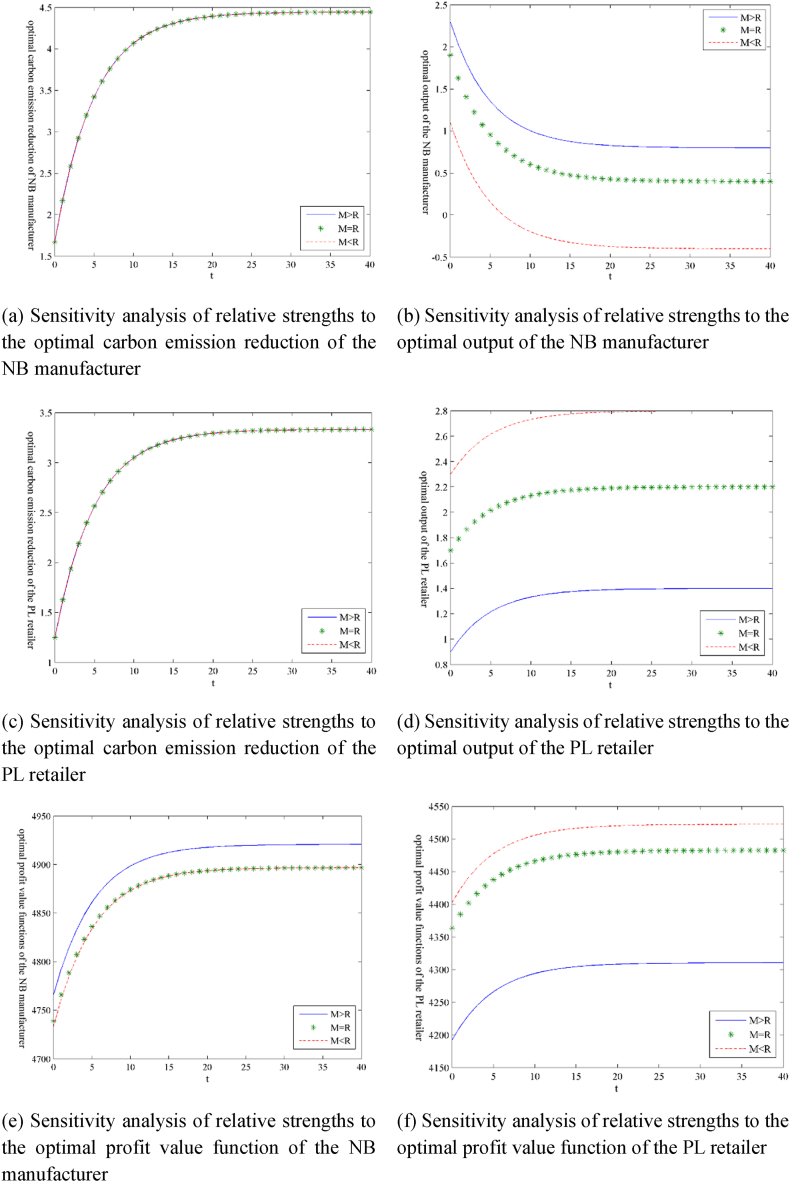
Sensitivity analysis of the relative strengths of game parties under the scenario ‘N’.

These results are shown in Fig. 5(ãf), where ‘M > R’, ‘M = R’, and ‘M < R’ indicate that the NB manufacturer is in a dominant position, the NB manufacturer and the PL retailer are peer to each other, and the PL retailer is in a dominant position, respectively.

Some results can be extracted from Fig. 5 as follows.

(i) The carbon emission reductions of the NB manufacturer and the PL retailer remain unchanged in the three cases because their carbon emission reductions are affected simply by the carbon trading guiding price and emission reduction efficiencies, as shown in Table 1.
(ii) For the NB manufacturer, the output and profit value functions reach maxima when ‘M > R’, followed by ‘M = R’, and attain minima when ‘M < R’. The NB manufacturer's profit value function has little difference between ‘M = R’ and ‘M < R’.
(iii) For the PL retailer, the output and profit value functions reach maxima when ‘M < R’, followed by ‘M = R’, and attain minima when ‘M > R’.

## 6 Conclusions, implications, and further studies

Using coopetition differential game theories, this study constructs dynamic coopetition game models between a PL retailer and an NB manufacturer under the carbon trading policy. The optimal feedback equilibria of the optimal carbon emission reductions and outputs between the NB manufacturer and the PL retailer are explored by using dynamic optimization control technology. The dynamic evolution trajectories of the variables and profit value functions are shown through numerical simulation. The sensitivities of key parameters and the relative strengths of the NB manufacturer and the PL retailer are analyzed.

We analyzed the dynamic evolution trajectories of state variables, decision variables, and profit value functions of the NB manufacturer and the PL retailer by numerical simulation in Subsection 5.3. We analyze further the trends as follows.

(i) The possible reason for differences in the optimal carbon reductions between the NB manufacturer and the PL retailer lies in the parameter settings. Generally speaking, the NB manufacturer has more experiences and more advanced emission reduction equipments than the PL retailer. Therefore, we set emission reduction efficiencies $\mu_{M}\left(=0.9\right)<\mu_{R}\left(=1.2\right)$ to represent that the NB manufacturer has a higher emission reduction efficiency so that the final emission reduction of the NB manufacturer is more significant than that of the PL retailer.
(ii) The possible reason for differences in the optimal outputs between the NB manufacturer and the PL retailer is that the PL product gradually shares the original product market after being on the market for a period, which leads to a lower output level for the NB product.
(iii) The possible reasons for differences in the optimal profit value functions between the NB manufacturer and the PL retailer are as follows: firstly, following the setting of initial parameters, we assume $h_{M}\left(=7\right)>h_{R}\left(=6\right)$, namely, the highest price that consumers are willing to pay for the NB product is greater than that for the PL product, and the NB manufacturer is in a higher position in the market compared with the PL retailer. Secondly, although the optimal output of the NB manufacturer decreases to a certain extent, it saves more carbon emission costs. It may even benefit due to the carbon emission right surplus.

From the sensitivity analysis results, we can get the following suggestions.

(i) The guiding price of carbon trading considerably affects the carbon trading market. It affects not only the steady-state value of the carbon trading price but also the steady-state values of carbon emission reductions, outputs, and profit value functions of the NB manufacturer and the PL retailer. The government must exercise great caution in setting a guiding price for carbon trading to ensure market stability.
(ii) The factors affecting steady-state values of outputs between the NB manufacturer and the PL retailer are exactly the same. But the effect trends on both are opposite. This reflects the competitive relationship between the two parties. Therefore, the NB manufacturer and the PL retailer should pay close attention to these factors to gain competitive advantages.
(iii) Besides the maximum retail price of PL product and carbon emission amount generated by unit PL product, the maximum retail price of NB product and the rate of return per unit NB product also affect the steady-state value of the retailer's profit value function with a tendency of ‘*E*-L+’ or ‘E + L-‘. The main reason may be that these factors affect the market shares of PL and NB products. The NB manufacturer and the PL retailer should improve these influencing factors based on mutual consultation. This point fully reflects competition and cooperation between both parties.
(iv) The relative strengths of both game sides have different effects on the outputs and profit value functions between the NB manufacturer and the PL retailer. It shows that consumers' recognition of products is significant.

Although our study has achieved some innovative results, it also has some limitations. Firstly, only the carbon trading price is considered as a constraint to construct the models. However, carbon trading market is a complex mechanism. For example, carbon trading price is generally affected by balance between supply and demand, and enterprises also have dishonest emission reduction behaviors. Therefore, future research may add carbon trading volume and incentive and punishment mechanism of the government into the constraint equations. Secondly, the PL retailer and the NB manufacturer are assumed rational and risk-neutral, but risk-seeking and risk-averse decision-makers also objectively exist in the real economic society. Some scholars have proved that supply chains can achieve better performances by considering human risk preferences [61]. Therefore, the risk preferences of NB manufacturers and PL retailers can be considered conditional assumptions in the future studies. Thirdly, a PL retailer and an NB manufacturer are assumed to be the decision-makers in this study. But consumers are important participants in the PL market and can affect carbon reduction decisions. Therefore, it would make sense if consumers’ behavior can be considered one of the constraints or consumers as one of the decision-makers to conduct models in future studies.

## Author contribution statement

Wenfang Yu: 1 - Conceived and designed the experiments; 2 - Performed the experiments; 3 - Analyzed and interpreted the data; 4 - Contributed reagents, materials, analysis tools or data; 5 - Wrote the paper. Guisheng Hou: 1 - Conceived and designed the experiments; 2 - Performed the experiments; 3 - Analyzed and interpreted the data; 4 -Contributed reagents, materials, analysis tools or data; 5 - Wrote the paper. Yu He: 1 - Conceived and designed the experiments; 2 - Performed the experiments; 3 - Analyzed and interpreted the data; 4 - Contributed reagents, materials, analysis tools or data; 5 - Wrote the paper. Baogui Xin: 1 - Conceived and designed the experiments; 2 - Performed the experiments; 3 - Analyzed and interpreted the data; 4 - Contributed reagents, materials, analysis tools or data; 5 - Wrote the paper.

## Funding statement

Dr. Baogui Xin was supported by 10.13039/501100010240National Planning Office of Philosophy and Social Science [21BJY206].

## Data availability statement

Data will be made available on request.

## Declaration of competing interest

The authors declare that they have no known competing financial interests or personal relationships that could have appeared to influence the work reported in this paper.
